# Outpatient treatment of acute poisoning by substances of abuse: a prospective observational cohort study

**DOI:** 10.1186/s13049-016-0268-6

**Published:** 2016-05-21

**Authors:** Odd Martin Vallersnes, Dag Jacobsen, Øivind Ekeberg, Mette Brekke

**Affiliations:** Department of General Practice, University of Oslo, Oslo, Norway; Department of Emergency General Practice, Oslo Accident and Emergency Outpatient Clinic, City of Oslo Health Agency, Oslo, Norway; Department of Acute Medicine, Oslo University Hospital, Oslo, Norway; Division of Mental Health and Addiction, Oslo University Hospital, Oslo, Norway; Department of Behavioural Sciences in Medicine, University of Oslo, Oslo, Norway

**Keywords:** Alcohol, Opioids, Benzodiazepines, Central stimulants, GHB, Triage

## Abstract

**Background:**

Procedures for the clinical assessment of acute poisoning by substances of abuse should identify patients in need of hospital admission and avoid hazardous discharges, while keeping the observation time short. We assess the safety of a systematic procedure developed at the Oslo Accident and Emergency Outpatient Clinic (OAEOC).

**Methods:**

All patients 12 years and older treated for acute poisoning by substances of abuse at the OAEOC were included consecutively from October 2011 to September 2012. Data were collected on pre-set registration forms. Information on re-presentations to health services nation-wide during the first week following discharge was retrieved from the Norwegian Patient Register and from local electronic medical records. Information on fatalities was obtained from the Norwegian Cause of Death Registry.

**Results:**

There were 2343 cases of acute poisoning by substances of abuse. The main toxic agent was ethanol in 1291 (55 %) cases, opioids in 539 (23 %), benzodiazepines in 194 (8 %), central stimulants in 132 (6 %), and gamma-hydroxybutyrate (GHB) in 105 (4 %). Median observation time was four hours. The patient was hospitalised in 391 (17 %) cases. Two patients died during the first week following discharge, both from a new opioid poisoning. Among 1952 discharges, 375 (19 %) patients re-presented at the OAEOC or a hospital within a week; 13 (0.7 %) with a diagnosis missed at the index episode, 169 (9 %) with a new poisoning, 31 (2 %) for follow-up of concomitant conditions diagnosed at index, and 162 (8 %) for unrelated events. Among the patients with missed diagnoses, five needed further treatment for the same poisoning episode, two were admitted with psychosis, one had hemorrhagic gastritis, another had fractures in need of surgery and four had minor injuries.

**Conclusion:**

The procedure in use at the OAEOC can be considered safe and could be implemented elsewhere. The high re-presentation rate calls for better follow-up.

## Background

Patients with self-inflicted poisoning often need hours of observation in emergency departments and are frequently admitted to hospital [1–3]. Many of these poisonings are by substances of abuse. Mostly, they are accidental overdoses of ethanol or drugs taken for purposes of intoxication or recreation. Some are suicide attempts [4, 5]. To identify the patients in need of hospital admission, keeping the observation time short, but long enough to avoid hazardous discharges, would be cost efficient and would help avoid crowding in emergency departments. Procedures have been suggested for different groups of poisoned patients [6–8].

The Oslo Accident and Emergency Outpatient Clinic (OAEOC), in Oslo, Norway, has for decades used a systematic clinical procedure for the assessment of patients presenting with suspected poisoning by substances of abuse [9]. This procedure is designed to enable the physician to identify patients in need of more intensive care than mere observation, and to identify conditions mimicking poisoning. The procedure demands less laboratory investigations than routinely are done in hospital emergency departments. Though not suitable for acute poisoning in general, the procedure could simplify management of patients with suspected poisoning by substances of abuse in hospital emergency departments. In 2008, only 14 % of the 1714 patients treated for accidental overdose with substances of abuse at the OAEOC were hospitalised [10]. Previous studies have shown that the procedure seems safe considering short-term mortality [10, 11]. However, no studies have addressed whether serious non-fatal conditions are overlooked among the patients discharged.

### Objectives

Our main aim was to assess the safety of the procedure in use at the OAEOC for treatment of acute poisoning by substances of abuse through charting mortality, cause of death and diagnosis at re-presentations to the OAEOC or any Norwegian hospital, within the first week following discharge from the OAEOC.

Further aims were to describe the acute poisonings by substances of abuse treated at the OAEOC and to identify factors associated with hospitalisation.

## Methods

The study was a prospective observational cohort study. The epidemiological results from the study have been published elsewhere [12].

### Setting

The Norwegian emergency care system has two levels. Patients cannot present directly to hospitals, but have to be assessed in primary care or by the ambulance service first. Primary care emergency services are provided by regular general practitioners during office hours and by local casualty clinics during nights and weekends. The OAEOC is the main casualty clinic in Oslo, serving the entire city at all hours. It is a combined emergency general practice service and trauma clinic, with limited diagnostic resources and observation capacity. It has about 200 000 consultations a year. The physicians are mostly registrars. It also contains a psychiatric emergency service, a 24-h observation unit and emergency social services. In most Norwegian cities, patients with acute poisoning by substances of abuse are treated in hospitals, but in Oslo the majority are treated at the Department of Emergency General Practice at the OAEOC [10–12]. Other casualty clinics in Oslo do not treat patients with acute poisoning to any significant extent. Oslo is the capital city of Norway and had a population of 613 285 as per 1 January 2012 [13].

The procedure in use at the OAEOC for the clinical assessment of patients presenting with suspected acute poisoning by substances of abuse was developed locally in the 1980s. It has regularly been revised. It consists of a basic clinical examination including vital signs, Glasgow Coma Scale (GCS) score and measurement of blood sugar level and peripheral capillary oxygen saturation (SpO_2_). The physician decides whether the patient should be hospitalised, observed at the OAEOC or discharged, based on standardised minimum clinical data gathered on an observational chart (Table 1). All patients presenting to the OAEOC are triaged using the Manchester Triage System (MTS), which gives the patients a priority for how soon a physician should see them [14]. After triage, patients with suspected acute poisoning by substances of abuse are examined and observed according to the observational chart. An assigned nurse observes the patient every 15–30 min. No toxicological screening tests are used, apart from breath analysis of ethanol. Point of care tests for haemoglobin, C-reactive protein and urine stick are available, as are electrocardiograms, CT head scans and regular X-rays. Blood gas levels are not measured. The main criterion for hospitalisation is finding a condition requiring acute medical or psychiatric care at hospital level. If the respiratory rate falls below ten per minute and SpO_2_ falls below 90 %, antidote should be given when opioid or benzodiazepine poisoning is suspected. When naloxone is administered, the patient should be observed for two hours in case of heroin poisoning, or hospitalised in case of long acting opioids. Patients with benzodiazepine poisoning in need of flumazenil should be hospitalised. Patients with respiratory depression caused by agents with no antidote or not responding to antidote should be hospitalised. Patients with suspected gamma-hydroxybutyrate (GHB) poisoning are often in a condition considered too unstable for observation at the OAEOC, with level of consciousness fluctuating from coma to agitation in need of sedation. Hence, the threshold for hospitalising these patients is low. Patients with hyperthermia due to central stimulant poisoning should be hospitalised, as should patients with psychosis. At the time of the study, comatose patients with a GCS score > 3, normal vital signs and nothing alarming on the minimum clinical examination were observed locally. The GCS threshold was later raised to a score > 6, based on the work of Forsberg et al. [15]. In either case, patients should be hospitalised if their level of consciousness is declining, or if they fail to regain consciousness within four hours. The maxim of the procedure is that conditions in need of treatment will show up in the clinical examination dictated by the observational chart.

**Table 1:** The standardised clinical set data gathered on the observational charts

Name	
Date of birth	
Date and time of presentation	
Who brought the patient?	
From where was the patient brought?	
Substances of abuse taken	
Time when taken	
Quantity taken	
What has happened? (free text)	
Previous medical history	
Seizures (yes/no)	
Diabetes (yes/no)	
Serious psychiatric disorder (yes/no)	
Known substance use (alcohol/opioids/other/none)	
Clinical status at presentation	
Respiratory rate	
Heart rate	
Blood pressure	
Temperature	
Blood glucose level	
SpO_2_	
Track marks (yes/no)	
External signs of injury (yes/no)	
Nystagmus (yes/no)	
Plantar reflexes (down/inverted)	
Pupil size (large/small/normal)	
Other information (free text)	
During observation time (fields provided for repeated observations)	
Glasgow Coma Scale score	
Pupil reaction to light (+/+)	
Respiratory rate	
SpO_2_	
Symmetric movement of arms and legs	
Medication given	
In ambulance	
At the casualty clinic	

The clinical assessment of acute poisoning by substances of abuse at the Oslo Accident and Emergency Outpatient Clinic is based on standardised minimum clinical data gathered on a pre-set observational chart

### Inclusion

All patients 12 years and older treated at the OAEOC for acute poisoning in which the main toxic agent was suspected to be a substance of abuse were included, irrespective of the intention behind the poisoning. All potential substances of abuse were included, encompassing alcohol, prescription drugs, illegal drugs and others. Patients treated for multiple conditions were included if the poisoning was serious enough to warrant treatment or observation. The period of inclusion was 1 year, from 1 October 2011 to 30 September 2012, to take seasonal variations into account. The physicians included patients consecutively. We regularly and systematically searched the patient lists in the electronic medical records and included any missed eligible patients.

### Participants

In total, 3139 cases of acute poisoning were registered. In 216 cases, the patient declined participation. In 406 cases, the suspected main toxic agent was not a substance of abuse, and the patient was excluded. Furthermore, in 174 cases, the patient did not have a Norwegian national identity number and was excluded, leaving 2343 included cases in 1731 patients.

### Data collection

Data were collected on registration forms and observational charts completed by the physicians and nurses treating the patients. We collected any missing information from the electronic medical records, along with data for patients included in the patient list searches.

Patients were identifiable by their unique Norwegian national identity number. Data on presentations to any Norwegian hospital during the first week following discharge from the OAEOC were retrieved from the Norwegian Patient Register (NPR), as were data concerning the hospital treatment of patients hospitalised from the OAEOC. The NPR registers all patient contacts in Norwegian hospitals and specialist health services. Data on fatalities and cause of death were obtained from the Norwegian Cause of Death Registry. Diagnoses were given as codes in the International Classification of Diseases and Related Health Problems 10th Edition (ICD-10). Data on new presentations at the OAEOC during the first week were gathered from the local electronic medical records.

### Outcome measures

The main outcome measures were fatalities and re-presentations to the OAEOC or any Norwegian hospital during the first week following discharge from the OAEOC. Time from discharge to re-presentation or death was calculated. Level of care and length of hospital stay were registered. Reason for re-presentation was categorised as missed diagnosis (including further treatment of the same poisoning), new poisoning, follow-up of concomitant condition diagnosed at index, and unrelated event. The categorisation was based on case notes in the electronic medical records at the OAEOC, and on diagnoses obtained from the NPR and the Norwegian Cause of Death Registry.

Additional measures were time and mode of presentation to the OAEOC, disposition and time of discharge, age, gender, toxic agents, reason for hospitalisation (more than one could be given), and the intention behind the poisoning. The physicians categorised intention as accidental overdose with substances of abuse, suicide attempt or mere accident/not self-inflicted. Poisonings with any degree of suicidal motivation or intentional self-harm were categorised as suicide attempts. The physicians diagnosed toxic agents based on all available information. The main toxic agent was defined as the one considered most toxic in the doses taken. Toxic agents were categorised as ethanol, opioids, benzodiazepines, central stimulants, GHB and other substances of abuse. Z-drugs were classified as benzodiazepines. Clinical observations included highest and lowest respiratory rate, lowest SpO_2_, lowest GCS score, heart rate, blood pressure, temperature, blood glucose level, track marks, external signs of injury, pupil reaction to light, nystagmus, plantar reflexes and symmetric movement of limbs. Breath analyser results, if available, were gathered from the patients’ electronic medical records. Any concomitant conditions and complications were registered by the physician treating the patient, along with medication given during observation or by ambulance personnel before arriving at the OAEOC, whether a CT head scan was done, and any involvement of other departments at the OAEOC. For hospitalised patients, we registered diagnoses at hospital and length of hospital stay.

### Statistics

Analyses were done in IBM SPSS version 21 (IBM Corp.). Mann–Whitney *U*-test was used when comparing continuous variables. Chi-square test was used to compare frequencies. Logistic regression analysis was used to estimate odds ratios for factors associated with hospitalisation. Relevant variables were first analysed one by one. The variables analysed were age, gender, main toxic agent, number of toxic agents, intention, naloxone treatment, clinical observations, complications and concomitant conditions. Ethanol was chosen as the reference group when estimating odds ratios for main toxic agents, as it was the largest group. Factors associated with hospitalisation in the univariate analyses with a significance level of *p* < 0.10, were included in the multivariate model.

### Ethics

The study was performed in accordance with the Helsinki declaration and approved by the Regional Committee South East for Medical and Health Research Ethics (REK nr 2010/1129-1) and the Oslo University Hospital Information Security and Privacy Office. Patients were included after having provided verbal consent.

## Results

There were 2343 cases of acute poisoning by substances of abuse included during 1 year, 1600 (68 %) were in males. Median age was 39 years among males and 30 years among females. The most frequent toxic agents were ethanol in 1291 (55 %) cases and opioids in 539 (23 %) (Table 2). In 761 (32 %) cases there were more than one toxic agent (range 1–5). Most poisonings, 2158 (92 %), were accidental overdoses with substances of abuse, 139 (6 %) were suicide attempts.

**Table 2:** Factors associated with hospitalisation—logistic regression analysis^a^

	n (%)	Hospitalised n (%)	Crude			Adjusted		
			Odds ratio	95 % CI	*p*	Odds ratio	95 % CI	*p*
Age								
26–50 years^b^	1207 (52)	245 (20)						
≤ 25 years	640 (27)	78 (12)	0.55	0.41–0.72	<0.001	0.75	0.53–1.0	0.090
> 50 years	496 (21)	68 (14)	0.62	0.47–0.84	0.002	1.2	0.83–1.7	0.348
Gender								
Males^b^	1600 (68)	288 (18)						
Females	743 (32)	103 (14)	0.73	0.57–0.94	0.013	0.74	0.55–1.0	0.052
Main toxic agent								
Ethanol^b^	1291 (55)	103 (8)						
Opioids	539 (23)	102 (19)	2.7	2.0–3.6	<0.001	**1.7**	1.1–2.5	0.014
Benzodiazepines	194 (8)	65 (34)	5.8	4.1–8.3	<0.001	**3.6**	2.2–5.8	<0.001
Central stimulants	132 (6)	42 (32)	5.4	3.5–8.2	<0.001	**2.5**	1.4–4.5	0.001
GHB	105 (4)	60 (57)	15.4	9.9–23.8	<0.001	**19.0**	11.3–32.0	<0.001
Other/Unknown	82 (3)	19 (23)	3.5	2.0–6.0	<0.001	**2.1**	1.1–4.2	0.026
Number of toxic agents			1.3	1.2–1.5	<0.001	0.89	0.75–1.1	0.204
Suicide attempt^c^	139 (6)	61 (44)	4.4	3.1–6.3	<0.001	**6.9**	4.2–11.4	<0.001
Treatment with naloxone^c^	198 (8)	48 (24)	1.7	1.2–2.4	0.003	1.1	0.72–1.8	0.581
Clinical observations^c^								
Respiratory depression^d^	287 (12)	86 (30)	2.5	1.9–3.3	<0.001	**2.4**	1.7–3.5	<0.001
Respiratory rate > 20/min	304 (13)	61 (20)	1.3	0.96–1.8	0.091	1.2	0.79–1.7	0.469
Heart rate ≥ 100/min	430 (18)	104 (24)	1.8	1.4–2.3	<0.001	**1.7**	1.2–2.4	0.002
Temperature ≥ 39.0 °C	11 (<1)	8 (73)	13.6	3.6–51.4	<0.001	1.8	0.34–9.3	0.503
Glucose ≤ 3.0 mmol/L	12 (1)	6 (50)	5.1	1.6–15.8	0.005	**6.5**	1.8–23.7	0.005
Neurological signs^e^	62 (3)	22 (35)	2.9	1.7–4.9	<0.001	**3.9**	2.1–7.3	<0.001
Hallucinations	59 (3)	31 (53)	5.9	3.5–10.0	<0.001	**9.6**	5.0–18.5	<0.001
Chest pain^c^	24 (1)	9 (38)	3.0	1.3–7.0	0.009	**5.6**	2.2–14.6	<0.001
Head injury^c^	217 (9)	24 (11)	0.60	0.38–0.92	0.021	0.97	0.59–1.6	0.888
Seizures^c^	42 (2)	17 (40)	3.5	1.9–6.6	<0.001	**5.7**	2.7–12.1	<0.001
Infection^c^	64 (3)	31 (48)	5.0	3.0–8.3	<0.001	**6.3**	3.5–11.3	<0.001
Other complications^c^	145 (6)	58 (40)	3.7	2.6–5.3	<0.001	**6.5**	4.2–10.0	<0.001
Lowest GCS score^f^								
15^b^	680 (29)	85 (13)						
10–14	1316 (56)	208 (16)	1.3	1.0–1.7	0.048	**2.0**	1.4–2.8	<0.001
8–9	223 (10)	49 (22)	2.0	1.3–2.9	0.001	**3.0**	1.8–4.9	<0.001
≤ 7	113 (5)	41 (36)	4.0	2.6–6.2	<0.001	**6.5**	3.7–11.6	<0.001
Total	2343 (100)	391 (17)						

Adjusted odds ratios for significant associations are shown in bold types

*CI* confidence interval, *GCS* Glasgow coma scale, *GHB* gamma-hydroxybutyrate

^a^ In the univariate analyses, the following variables were not significantly associated with hospitalisation (*p* ≥ 0.10): systolic blood pressure ≥180 (*n* = 12, 4 (33 %) admitted); systolic blood pressure ≤ 90 (*n* = 117, 21 (8 %) admitted); temperature ≤ 34.0 °C (*n* = 34, 8 (23 %) admitted); glucose ≥ 14.0 mmol/L (*n* = 19, 5 (26 %) admitted); track marks (*n* = 375, 60 (16 %) admitted); other injuries (*n* = 171, 29 (17 %) admitted); and breath analysis alcohol level (analysed in 805 (34 %) patients, median 190 mg/dL in hospitalised patients, 210 mg/dL in discharged patients)

^b^ Reference group

^c^ The reference groups are not suicide attempt/no treatment with naloxone/observation or complication not recorded (reference groups not shown)

^d^ Respiratory rate < 10/min, SpO_2_ < 90 %, or in need of respiratory support

^e^ Comprises nystagmus, inverted plantar reflex, asymmetric pupil reaction to light or asymmetric movement of limbs

^f^ Eleven cases missing

Two patients died within the first week following discharge, both from a new poisoning. One died from an unintentional heroin overdose at least 16 h after being discharged to the Emergency Social Services after five hours of observation for a possible suicide attempt with heroin, benzodiazepines and anabolic steroids. The other died from an unintentional methadone overdose the 5th day, having self-discharged after nine hours of observation for an accidental overdose with buprenorphine and benzodiazepines which was treated with naloxone 0.4 mg intramuscularly shortly after presentation.

**Fig. 1:**
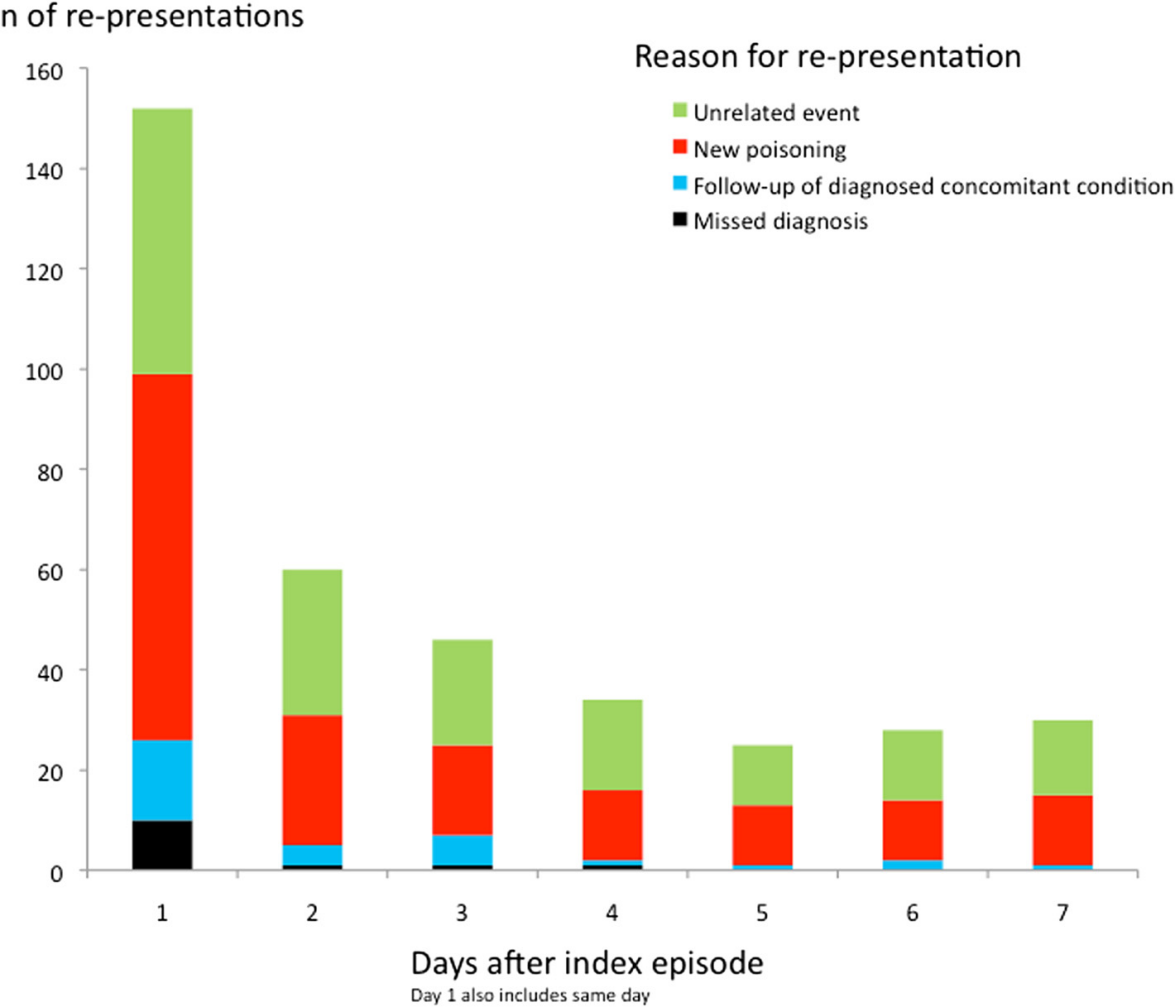
Re-presentations during the first week following discharge from the Oslo Accident and Emergency Outpatient Clinic

In 1952 (83 %) cases, the patient was discharged. Among them, 375 (19 %) re-presented at the OAEOC or a hospital during the first week following discharge (Fig. 1); 13 (0.7 %) with a diagnosis missed at the index episode, 169 (9 %) with a new poisoning, 31 (2 %) for follow-up consultations concerning concomitant conditions diagnosed at index, and 162 (8 %) for reasons not related to the index episode. The age range of patients with missed diagnoses was 14–49 years, and 9/13 (69 %) were males (Table 3). Among the re-presenting patients, 88/375 (23 %) had self-discharged. Among the patients re-presenting with a new poisoning, 46/169 (27 %) had self-discharged.

**Table 3:** Patients with missed diagnoses at index episode

Patient gender and age	Diagnosis at re-presentation	Level of care at re-presentation	Time from discharge to re-presentation	Toxic agents at index^a^	Treatment, disposition and observation time (hours:minutes) at index
M 38	Increasingly somnolent from same poisoning, probably also long-acting opioid, concomitant compartment syndrome	4 days in medical department	1 h	Heroin	Naloxone 1.6 mg by ambulance Discharged to addiction clinic 5:25
M 47	Brought back for same poisoning	Outpatient at the OAEOC	1 ½ hours	Ethanol	Self-discharged 0:45
F 21	Brought back for same poisoning	Outpatient at the OAEOC	3 h	Ethanol, fluoxetine	Self-discharged 1:00
M 14	Increasingly somnolent from same poisoning	2 days in pediatric department	3 h	Heroin, benzodiazepines	Discharged to Child Welfare Services 2:10
F 40	Psychosis	7 days in psychiatric ward	Same day	Heroin, amphetamine	Discharged, no follow-up 8:15
M 34	Psychosis	4 days in psychiatric ward	Same day	Heroin	Referred to psychiatric outpatient clinic 5:40
F 28	Concussion, sprained ankle	Outpatient at the OAEOC	Same day	Ethanol	Discharged, no follow-up 5:55
M 29	Contusions	Outpatient at the OAEOC	Same day	Ethanol, amphetamine	Discharged, no follow-up 4:15
M 21	Increasingly somnolent, possibly from same poisoning, if so also with sedatives	1 day in medical department	Next day^b^	Ethanol	Discharged to police custody 0:45
M 20	Concussion, contusions	Outpatient at the OAEOC	Next day	Ethanol	Discharged to Emergency Social Services 4:10
F 49	Haemorrhagic gastritis	5 days in medical department	2nd day	Ethanol	Referred to social services 10:35
M 46	Non-dislocated fracture of zygomatic bone	Outpatient at the OAEOC	3rd day	Ethanol	Discharged, no follow-up 6:05
M 44	Fracture of elbow and wrist	5 days in surgical department	4th day	Ethanol, cannabis	Self-discharged 2:15

*OAEOC* Oslo Accident and Emergency Outpatient Clinic

^a^All were accidental overdoses with substances of abuse

^b^At least 18 h later

The patient was brought by ambulance in 1431 (61 %) cases, by the police in 393 (17 %), by addiction outreach services in 96 (4 %), by companions in 141 (6 %), by others in 86 (4 %), and presented on their own in 196 (8 %) cases. Clinical observations, complications and concomitant conditions are shown in Table 2. A CT scan was done in 144/217 (66 %) patients with concomitant head injury, eight were positive. Medical treatment was given in 402 (17 %) cases (Table 4). Naloxone was given in 198 (8 %) cases; intramuscularly in 123/198 (62 %), intravenously in 6/198 (3 %), and both in 51/198 (26 %). Naloxone was given by the ambulance service in 138/198 (70 %) cases, at the OAEOC in 45/198 (23 %), and both in 15/198 (8 %). Mean number of doses was 1.4 (range 1–4), mean total dose was 0.8 mg (range 0.2–2.4). No patients died at the OAEOC.

**Table 4:** Specific treatment given at the OAEOC

	Total n (%)	Hospitalised n (%)
Medical treatment	402 (17)	103 (26)
Naloxone	198 (8)	48 (24)
Paracetamol	57 (2)	10 (18)
Thiamine	52 (2)	11 (21)
Intravenous fluids	51 (2)	17 (33)
Valproate	42 (2)	8 (19)
Alimemazine	32 (1)	6 (19)
Oxygen	30 (1)	20 (67)
Diazepam	21 (1)	14 (67)
Nitrazepam	19 (1)	5 (26)
Metoclopramide	16 (1)	2 (13)
Haloperidol	15 (1)	5 (33)
Active charcoal	8 (<1)	3 (38)
Flumazenil	3 (<1)	2 (67)
Resuscitated at the OAEOC	1 (<1)	1 (100)
Other	82 (3)	21 (26)
Departments involved besides the emergency general practice service		
Trauma clinic	223 (10)	28 (13)
Psychiatric emergency service	64 (3)	8 (13)
Emergency social services	500 (21)	11 (2)
24 h observation unit	102 (4)	17 (17)
Total	2343 (100)	391 (17)

*OAEOC* Oslo Accident and Emergency Outpatient Clinic

In 391 (17 %) cases the patient was hospitalised; 321/391 (82 %) to medical departments, 23/391 (6 %) to other somatic departments, and 49/391 (13 %) to psychiatric wards. The factors most strongly associated with hospitalisation were GHB poisoning (adjusted odds ratio (AOR) 19.0, 95 % confidence interval (CI) 11.3–32.0), hallucinations (AOR 9.6, 95 % CI 5.0–18.5), suicide attempt (AOR 6.9, 95 % CI 4.2–11.4), hypoglycaemia (AOR 6.5, 95 % CI 1.8–23.7), and GCS score ≤ 7 (AOR 6.5, 95 % CI 3.7–11.6) (Table 2). Median observation time at the OAEOC was 3 h 55 min (interquartile range (IQR) 2 h 10 min–5 h 20 min), but significantly shorter for patients hospitalised, median 1 h 50 min (IQR 1 h 00 min–4 h 10 min), than for patients discharged from the OAEOC, median 4 h 15 min (IQR 2 h 35 min–5 h 25 min) (*p* < 0.001). Hospitalisation of patients with GHB poisoning, low GCS score or hyperthermia occurred early, while hospitalisation of patients with hypoglycaemia or infection occurred late. The main reason for hospitalisation was the poisoning itself in 206 (53 %) cases, and unclarified condition in 113 (29 %) cases (Table 5). Median length of hospital stay was 1 day (IQR 0–2, range 0–191), 129 patients (33 %) stayed more than 24 h. One patient with ethanol poisoning, resuscitated at the OAEOC after cardiac arrest subsequent to aspiration during transport, died from respiratory failure due to pneumonitis after 25 days in hospital.

**Table 5:** Reasons for hospitalisation, and diagnoses at hospital

	Reason given for hospitalisation by physician at the OAEOC					
Diagnosis at hospital	Poisoning n (%)	Injury n (%)	Other somatic condition n (%)	Psychiatric condition n (%)	Unclarified condition n (%)	Total n (%)
Poisoning	171 (83)	2 (20)	27 (34)	25 (45)	63 (56)	236 (60)^a^
Injury	-	7 (70)	5 (6)	-	3 (3)	14 (4)^a^
Infection	6 (3)	-	13 (16)	1 (2)	5 (4)	21 (5)^a^
Other somatic	17 (8)	1 (10)	25 (31)	2 (4)	20 (18)	57 (15)^a^
Psychiatric	11 (5)	-	2 (3)	14 (25)	5 (4)	27 (7)^a^
Substance abuse	11 (5)	1 (10)	10 (13)	12 (21)	14 (12)	41 (10)^a^
Did not present	-	-	-	4 (7)	4 (4)	8 (2)^a^
Total	206 (100)^a^	10 (100)^a^	80 (100)^a^	56 (100)^a^	113 (100)^a^	391 (100)^a^

*OAEOC* Oslo Accident and Emergency Outpatient Clinic

^a^Numbers and percentages may add up to more than total (100) as some patients were hospitalised for several reasons and some received more than one diagnosis at the hospital

The patients who declined participation did not significantly differ in age (*p* = 0.81) or gender (*p* = 0.75) from the rest. In the 174 cases excluded due to lack of a Norwegian national identity number, the patients were younger (median age 27, *p* < 0.001) and more often male (81 %, *p* = 0.001) than in the included cases. There were no significant differences in main toxic agents (*p*-values ranging from 0.064 to 0.56), apart from a smaller proportion of central stimulants (2 %, *p* = 0.042). There were no significant differences in intention (*p* = 0.18), observation time (*p* = 0.50) or proportion hospitalised (*p* = 1.00). Apart from larger proportions with low GCS scores (11 % with GCS ≤ 7, *p* = 0.001, and 20 % with GCS 8–9, *p* < 0.001) and tachypnoe (21 %, *p* = 0.006), there were no significant differences in the clinical measures listed in Table 2 (*p*-values ranging from 0.11 to 1.00).

## Discussion

The management of ethanol, opioid, benzodiazepine, GHB and central stimulant poisoning is well established [16–22]. There has been some controversy about how long patients need observation, especially patients treated with naloxone for opioid overdose. Christenson et al. developed a set of criteria for discharging patients one hour after naloxone administration [6]. However, recurrence of respiratory depression in poisoning with long acting opioids may occur outside this time frame, and current practice at the OAEOC is based on a review by Clarke et al. recommending an observation time of two hours [23].

Patients observed at the OAEOC are hospitalised if they do not regain full consciousness within 4 h. The 4-h limit was set as patients poisoned by ethanol or heroin, the two most frequent toxic agents, should be awake and recovering by this time. If not awake or if confusion persists, other conditions must be suspected, most of them beyond the scope of what can be managed in the outpatient setting [15, 16, 24].

The three patients re-presenting increasingly somnolent seem to have been discharged too early. Even though one of them was observed for five and a half hours and was alert at discharge, he was somnolent one hour later. They were all discharged into someone’s custody. Psychotic symptoms during the initial observation may have been masked by heroin in the two patients re-presenting with psychosis. The self-discharging patients were advised to stay for further observation, unless they left without notice. The haemorrhagic gastritis and the minor injuries were overlooked.

The observational chart (Table 1) is not a treatment algorithm, but more of a checklist for the clinical examination of patients with suspected acute poisoning by substances of abuse. Other monitoring systems, like the Rapid Emergency Triage and Treatment System (RETTS) [25] and the modified Early Warning Score (MEWS) [26], could have been applied. However, the locally developed procedure has the advantage of being specifically tailored for patients with suspected acute poisoning by substances of abuse. In addition, supervision is close, with repeated observations every 15–30 min, to identify patients whose condition is deteriorating.

Though figures are not available, the treatment of patients with acute poisoning by substances of abuse at the OAEOC is probably less expensive than in hospital emergency departments. The observation time is short, with three out of four patients staying less than five and a half hours, and fewer supplementary investigations are done. In addition, outpatient treatment at the OAEOC means less crowding in Oslo hospital emergency departments, allowing them to focus their resources on the more severely sick patients.

In our study, less than one percent of nearly 2000 discharges needed further treatment for the same poisoning or had a missed diagnosis. Two patients died from a new overdose within one week. In two previous 1-year studies of acute poisoning at the OAEOC, in 2003 and 2008, the same methods were used, except that re-presentations were not traced [10, 11]. In 2008, among 1865 discharges, nine patients died during the first month; one from possible recurrence of opioid toxicity, one committed suicide, five died of a new overdose, and two from unrelated causes [10]. In 2003, among 801 discharges, no patients died during the first week [11]. No patients with poisoning died at the OAEOC in any of the studies. In our study, one patient hospitalised from the OAEOC died in hospital. During the two previous study periods, a total of 14 patients died in Oslo hospitals from acute poisoning by substances of abuse [27, 28]. We do not know whether these patients were brought directly to hospital by ambulance, or if any of the in-hospital fatalities could have been precipitated by delay at the OAEOC. Furthermore, there are uncertainties concerning the fate of the excluded patients in our study. Still, it is our opinion that the results of the three studies justify considering the observational procedure at the OAEOC to be safe enough.

The deaths from new overdoses shortly after discharge, and the high number of new non-fatal overdoses, are signs of hazardous substance use among these patients. This is in line with other studies showing that an acute poisoning is a major risk factor for new poisoning and death [29–31]. Consequently, the acute poisoning episode would seem a suitable time for intervention, e.g. brief interventions before discharge, ensuring follow-up by general practitioner, and/or referral to the specialist health services. Still, during the study period, 36 % of the patients treated for acute poisoning were discharged without follow-up, an additional 13 % self-discharged [12].

### Strengths and limitations

Our study encompassed the vast majority of patients with acute poisoning by substances of abuse treated at the OAEOC. The consecutive inclusion and the systematic searches in the electronic patient lists ensured the inclusion of practically all the eligible patients. In addition to the 174 patients without a Norwegian national identity number, 216 patients declined to participate. We do not think these patients differ from the included patients in ways that would have an impact on our main results.

The patients’ unique Norwegian national identity number allowed us to track re-presentations at hospitals nation-wide. Other casualty clinics in or around Oslo do not treat acute poisoning to any significant extent. However, in 2003, 721 patients were left on scene after treatment by the ambulance service in Oslo, among them 367 given naloxone for opioid poisoning [11]. We believe the figures to have been in the same range in 2012. Unfortunately, we did not have access to the ambulance medical records. Thus, we probably missed some re-presentations among patients only treated by the ambulance service the first week following discharge. Still, any fatalities and patients with conditions severe enough to be in need of treatment at the OAEOC or a hospital, would show up in our data.

In 2008, nearly 200 patients with accidental overdoses with substances of abuse bypassed the OAEOC and were brought directly to hospital by the ambulance service due to the severity of the poisoning [10, 28]. We expect that these patients would have been sent on to hospital had they been brought to the OAEOC.

Diagnosis of toxic agents was based on self-report and clinical examination. No laboratory confirmation was done. While we do not know which toxic agents our patients had taken, the categories in our study refer to toxidromes that to some extent are clinically distinguishable. It is likely that new psychoactive substances may have been diagnosed as amphetamine or cannabis [12]. The clinical picture often associated with GHB poisoning, with fluctuating level of consciousness and intermittent agitation, may also be the result of a mixture of central stimulants and central depressants. Furthermore, a Danish study sampling blood from patients with heroin overdose, found that nearly all the patients had taken a variety of additional substances of abuse [32]. Still, our results are based on diagnoses and decisions made in a real clinical situation. We think this adds value to their generalizability. It also reflects current best practice in toxicology in which patients are managed based on clinical assessment. In most cases, treatment decisions have to be made before reliable laboratory confirmation is available. Along these lines, we found that the patient’s clinical condition had greater impact on hospitalisation than which toxic agents were diagnosed, as odds ratios for hospitalisation were greater in the multivariate than in the univariate analysis for several clinical observations and for suicidal intent, while for toxic agents, except GHB, they were smaller (Table 2).

## Conclusion

We consider the OAEOC procedure for the clinical assessment of acute poisoning by substances of abuse to be safe enough. It may be implemented elsewhere. The information gathered on the observational chart gives clinicians the necessary basis for deciding whether the patient can either be discharged, stay for observation or requires treatment or further investigations, locally or at hospital level. The high re-presentation rate during the first week—mostly due to a new poisoning—calls for better follow-up.
